# Increased Sulphur Amino Acids Consumption as OH-Methionine or DL-Methionine Improves Growth Performance and Carcass Traits of Growing-Finishing Pigs Fed under Hot Conditions

**DOI:** 10.3390/ani12172159

**Published:** 2022-08-23

**Authors:** Caio Abércio da Silva, Cleandro Pazinato Dias, Marco Aurélio Callegari, Kelly Lais de Souza, José Henrique Barbi, Naiara Simarro Fagundes, Dolores I. Batonon-Alavo, Luciana Foppa

**Affiliations:** 1 Department of Animal Production, Center of Agrarian Science, State University of Londrina, Londrina 86057-970, PR, Brazil; lufoppa@yahoo.com.br; 2 Akei Animal Research, Estrada Vicinal Fartura—Areias, Km 3|Três Saltos, Fartura 18870-000, SP, Brazil; cleandropazinato@uol.com.br (C.P.D.); marcoacallegari@gmail.com (M.A.C.); tecnico@akei.agr.br (K.L.d.S.); 3 Adisseo France S.A.S., 10, Place du Général de Gaulle, 92160 Antony, France; jose.barbi@adisseo.com (J.H.B.); naiara.fagundes@adisseo.com (N.S.F.); dolores.batonon-alavo@adisseo.com (D.I.B.-A.)

**Keywords:** heat stress, methionine, hydroxy-methionine, pigs, performance

## Abstract

**Simple Summary:**

Our results suggest that increased methionine consumption by growing- finishing pigs leads to improvement of performance and carcass quality. Moreover, supplementation with OH-methionine improved the loin depth. Taken together, our findings indicate that it might be necessary to update the recommendations for methionine for growing-finishing pigs raised under tropical conditions.

**Abstract:**

This study aimed to evaluate the impact of DL-Methionine (DL-Met) or OH-Methionine (OH-Met) when supplemented beyond the usually accepted requirements in sulfur amino acids (SAA) on the performance and carcass traits of growing-finishing pigs. Two hundred mixed sex pigs were distributed in a randomized block (body weight and sex), under a 2 × 2 factorial design with two methionine sources, DL-methionine, or OH-Methionine and two methionine doses (100% SAA or 120% of the SAA level present in the control). Diets were formulated to meet amino acids recommendations of the Brazilian Tables for Poultry and Swine (2017), except for SAA, which varied with the methionine doses. Daily feed intake, daily weight gain, and feed conversion were evaluated. Moreover, the carcasses were measured electronically for fat thickness (FT), longissimus dorsi muscle depth (LD), and lean meat (%). During the growing phase II (92 till 122 days of age), daily feed intake (*p* < 0.001) and daily weight gain (*p* < 0.05) increased with the high SAA levels. High SAA levels also provided greater daily weight gain during the entire period of the trial (0.90 versus 0.86 kg; *p* < 0.05) No significant interaction was observed between the methionine source and the SAA level for any carcass traits. However, animals that received OH-Met had greater (*p* < 0.05) loin depth (58.37 versus 55.21 mm) and those that received higher doses of methionine presented heavier (*p* < 0.05) carcass weight (78.16 versus 74.70 kg), and more (*p* < 0.05) lean meat weight (43.69 versus 41.90 kg). Taken together, these results demonstrated that supplementation of high sulfur amino acids levels under hot conditions provided heavier carcasses and more lean meat.

## 1. Introduction

Methionine is a sulphur-containing amino acid (TSAA—total sulphur amino-acid), considered the third limiting amino acid used in commercial swine diets after lysine and threonine [1]. However, it may be also the second limiting when protein sources with high lysine content are used [2], explaining the supplementation of synthetic methionine sources. Methionine is also the precursor of creatine, carnitine, and polyamine, which are essential for cell differentiation and proliferation [3]. TSAAs are included in the official nutritional requirements of the NRC [2], ARC [4], and Brazilian Tables for Poultry and Swine [5] for swine, but these requirements are not up-to-date and may be little in line with the following examples of progress in genetics. According to Conde-Aguilera et al. [6] and Conde-Aguilera et al. [7], a TSAA deficient diet in growing pigs can alter their protein deposition rate and tissue protein composition, thus affecting their carcass and meat yield. On the other hand, TSAA deficiency can modify the amino acid profile of tissues, glutathione-redox balance, and muscle fiber type [8,9].

Synthetic methionine can either be supplied in the form of L-Methionine, which is the biologically active form that initiates protein synthesis, or in the form of DL-Methionine (DL-Met) or 2-Hydroxy-4MethylThio-Butanoic acid (OH-Met) as these are converted into L-Methionine [10]. Previous research indicates that OH-Met is absorbed in the upper gastrointestinal tract for broilers and pigs, while DL-Met is mainly absorbed in the jejunum and only is completed in the final portion of the ileum [11,12].

Most studies regarding different sources of methionine in pigs focus on the effects on piglets in the nursery phase [13] and only a few have been carried out to compare the effects of synthetic methionine sources on performance in the growing and finishing phases.

Another important factor to consider is that high temperatures tend to alter nutrient utilization patterns, carcass quality, and performance. Working with chickens exposed to high temperatures, Willemsen et al. [14] observed a significant increase in hepatic total glutathione (GSH) and oxidized glutathione (GSSG) levels, regardless of the supplemental source. However, the hepatic ratios of reduced GSH to total GSH and reduced GSH to GSSG were highest in chickens supplemented with OH-Met compared with DL-Met, preventing the growth-depressing effects. This is of great concern, considering the rate of expansion in swine production in the tropics. Therefore, finding out the real impact of these conditions on dietary supplements has become crucial.

In view of the above, the aim of this research is to evaluate the impact of methionine sources (DL-Met or OH-Met) when supplied at the currently determined requirement and above in sulphur amino acids on the growth performance and quality of carcass in the growing-finishing pigs under hot conditions.

## 2. Materials and Methods

### 2.1. Ethics Statement

This study was carried out following the recommendations from the Guide for the Care and Use of Laboratory Animals of the National Animal Experimentation Control Council of Brazil (CEUA). The trial was approved by the Ethics Committee of Animal Experiments of Akei Animal Research (protocol number 012.18).

### 2.2. Animals, Treatments, and Measurements

**Figure 1:**
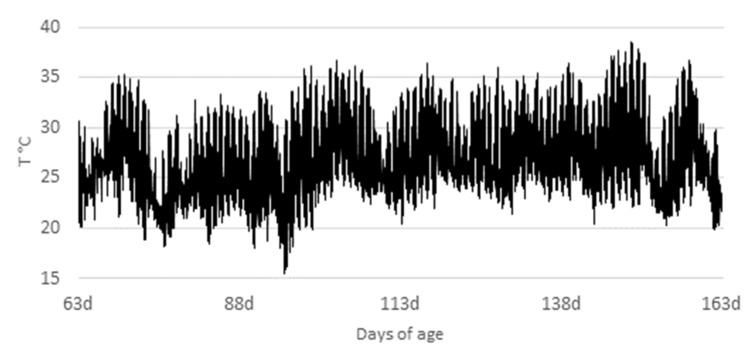
Evolution of temperature (°C) in the facility during the experimental period.

**Figure 2:**
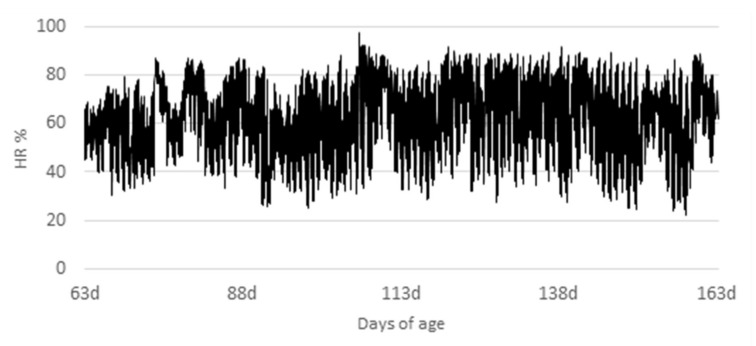
Evolution of relative humidity (%) in the facility during the experimental period.

The animals used in the study were obtained from a commercial farm after the nursery phase. They were housed in a 50-stall brick barn (5.85 m^2^/stall) with semi-slatted plastic floors equipped with a (0.35 m) Dutch feeder and adjustable nipple drinkers. The maximum and minimum temperatures and relative air humidity were measured throughout the experimental period with a data logger (Hobo^®^; accuracy ± 0.2 °C; data logger temperature/RH: Onset^®^; Bourne, MA, USA) and are presented in Figure 1 and Figure 2.

Two hundred pigs (PIC 337 × Camborough), half barrows and half females, were used in this study. The animals began the experiment at 63 days old with an average body weight of 20.427 ± 1.997 kg. They were subjected to four treatments for 97 days, until 160 days old. At the beginning of the trial, animals were distributed in four blocks (according to the initial body weight and sex), with 10 replicates each. A replicate was a floor pen of five pigs of the same sex (barrows or females).

The experimental treatments consisted of a 2 × 2 factorial design with two methionine sources, DL-Met (99% of methionine, powder) and OH-Met (88% of methionine, liquid); and two SAA levels (100% and 120% of the SAA level present in the control). DL-Met and OH-Met were supplemented on equimolar basis in all treatments at the expense of corn. Diets were formulated to meet amino acids recommendations of the Brazilian Tables for Poultry and Swine [5] for high performance pigs, except SAA, the levels of which varied depending on the treatments.

The experimental diets were formulated based on corn, soybean meal, wheat, and DDGS (distillers’ dried grains with soluble) (Table 1). Animals were fed during four rearing phases: grower I (63–91 days of age); grower II (92–112 day); finisher I (113–140 days); and finisher II (141–160 days). Feed and water were provided ad libitum throughout the experimental period. The nutritional composition and calculated nutritional values of the experimental feeds are presented on Table 1.

The following parameters were evaluated for each rearing period and on the whole experimental period: daily feed intake, daily weight gain and feed conversion, as well individual body weight. Additionally, methionine consumption per pig per day (based on calculated values of feed intake) was evaluated according to the experimental phases and considering the whole experimental period. At the end of the experiment, all animals were taken to a commercial slaughterhouse. Before loading and transport, the animals were fasted from solids for 12 h and received only water until the transport to slaughter. After slaughtering and evisceration, the carcasses were cut longitudinally and refrigerated at 2 ± 1 °C for 24 h in a cold chamber. Each carcass was measured electronically (using Hennessy Grade Probe, Hennessy Grading Systems, Auckland, NZ) for fat thickness (FT), L. dorsi muscle depth (LD), measured at point P2, and lean meat (%). Lean meat weight (kg) was also calculated based on the measured carcass weight multiplied by the percentage of lean meat. To estimate the percentage of processable lean meat (PLM), an equation, based on the Hennessy standard, was used: PLM = 61.33 − (0.76 × LD) + (0.1 × FT).

### 2.3. Feed Analyses

Dry matter and gross energy in experimental feeds were analyzed based on AOAC [15] methods. For amino acid analysis, the proteins of feeds were previously hydrolyzed with 6 N hydrochloric acid for 24 h. After, the amino acids were released in acid hydrolysis reacted with Phenylisoethylcyanate (PITC), separated by reversed phase HPLC and detected by U.V. at 254 nm. Quantification was done by multilevel internal calibration, with the aid of Alpha-Aminobutyric acid (AAAB) as an internal standard.

**Table 1:** Composition and calculated nutritional values of the experimental diets.

Ingredients (%, As-Fed)	Grower I (63–91 Days Old)		Grower II (92–112 Days Old)		Finisher I (113–140 Days Old)		Finisher II (141–160 Days Old)	
	100% SAA	120% SAA	100% SAA	120% SAA	100% SAA	120% SAA	100% SAA	120% SAA
Corn	53.071		58.325		62.518		66.012	
Soybean meal	18.967		14.493		10.834		7.638	
Soybean oil	4.410		3.956		3.625		3.403	
Wheat grain	12.500		12.500		12.500		12.500	
DDGS	7.500		7.500		7.500		7.500	
Dicalcium phosphate	1.288		1.060		0.920		0.864	
Limestone	0.826		0.764		0.726		0.716	
L-lysine	0.535		0.537		0.541		0.545	
NaCl	0.218		0.193		0.171		0.160	
Sodium sulfate	0.183		0.180		0.180		0.179	
Vitamin-Mineral Premix *	0.200		0.200		0.200		0.200	
Methionine source (DL-Met/OH-Met) *	0.081/0.091	0.145/0.163	0.079/0.089	0.130/0.146	0.083/0.093	0.141/0.159	0.050/0.057	0.099/0.112
L-threonine	0.183		0.171		0.162		0.155	
L-tryptophan	0.055		0.058		0.060		0.062	
L-valine	0.059		0.058		0.060		0.061	
Calculated composition (%)								
Metabolizable energy (kcal/kg)	3350		3350		3350		3350	
Crude protein	16.97		15.34		14.00		12.82	
Fiber	2.89		2.69		2.52		2.37	
SID Lys	1.07		0.96		0.88		0.81	
SID Met equivalent	0.32	0.38	0.30	0.36	0.29	0.35	0.24	0.29
SID Met + Cys equivalent	0.63	0.76	0.58	0.71	0.56	0.68	0.46	0.56
SID Thr	0.69		0.63		0.57		0.53	
SID Trp	0.21		0.19		0.18		0.16	
SID Val	0.74		0.67		0.61		0.56	
Available phosphorus	0.36		0.31		0.28		0.27	
Calcium	0.73		0.63		0.57		0.55	
Chloride	0.18		0.17		0.15		0.15	
Sodium	0.19		0.18		0.17		0.16	
Potassium	0.48		0.44		0.42		0.39	
Dietary Electrolyte Balance	153		143		135		127	

* Methionine sources as DL-methionine 99% and OH-methionine (DL-HMTBA) as 88%. Levels per kilogram of product: Vit. AD3 0,000 kUI; Vit. A 6000.000 kUI; Vit. D3 1500.000 kUI; Vit E 15,000.000 mg; Vit K3 (Menadione); 1500.000 mg Vit. B1 (Tiamine); 1350.000 mg Vit.B2 (Riboflavine) 4000.000 mg; Vit. B6 (Piridoxine) 2000.000 mg; Vit. B12 (Cianocobalamine) 20.000 mg; Niacin (Ac.Nicotiinico) 20,000.000 mg; Pantothenic Ac 9350.000 mg; Ác. Pholic Ac 600.000 mg; chelated Selenium 300,000 mg; Biotin 80,000 mg; Copper 10,000.000; Iron 100,000.000 mg; Manganese 40,000.000 mg; Cobalt 1000.000 mg; Iodine 1500.000 mg; Zinc 100,000.000 mg. SID = standardized ileal digestibility.

### 2.4. Statistical Analysis

Animal growth performance data and carcass traits were subjected to two-way analysis of variance using the methionine source and the SAA level as main variables, in R program (R version 3.5.1). The individual body weight was also analyzed as mentioned above. The animal was considered the experimental unit for individual body weight and carcass traits whereas the floor pen was considered as the experimental unit for the collective growth performance criteria. Significance was based on *p*-value equal to or less than 0.05, and a *p*-value between 0.05 and 0.10 was considered as a trend.

## 3. Results

Temperature and relative humidity varied widely between day and night, and throughout the experiment. Average temperature was about 27.12 ± 4.51 °C and relative humidity was 61.72 ± 5.65%. Temperature was above the thermoneutrality, which is recommended to be around 16 to 27 and 10 to 24 °C for growing and finishing pigs, respectively [15]. These results show that the animals were raised in an environment with the characteristics of a tropical climate.

The results of feed analysis (Table 2) show that the levels of dry matter and gross energy are according to applied treatments. Additionally, the levels of synthetic methionine added were similar, independent of the sources used, as well as the other amino acids.

The calculated methionine and methionine + cysteine consumption is shown on Table 3. For both amino acids, consumption per pig per day in all phases and considering the whole experimental period was significatively different (*p* < 0.05), favoring the high-level SAA treatment.

### 3.1. Similar Growth Performance between Methionine Sources

The results of the collective growth performance are presented in Table 4. These showed no interaction effects (Met source x SAA level) for any parameters for all rearing periods. Animals which received 120% of SAA presented the highest daily feed intake (DFI) and daily weight gain (DWG) during the growing phase II (2.252 versus 2.096 kg/d, *p* = 0.006 and 1.009 versus 0.947 kg/d, *p* = 0.026, respectively), and the finisher phase I (2.597 versus 2.494 kg/d, *p* = 0.087 and 0.968 versus 0.914 kg/d, *p* = 0.090, respectively), and also highest DWG for the total period (0.908 versus 0.863 kg/d, *p* = 0.030). Met source showed no effect on collective growth performance.

Considering the individual live weight of the pigs, there was no interaction effect for this parameter in any phase (Table 5). However, animals fed with higher dose of methionine (120%) were heavier at 112 days (*p* = 0.051), at 140 days of age (*p* = 0.055), and at the end of the trial (*p* = 0.055; 163 days of age).

### 3.2. Effects on Carcass Quality Traits

There were no interaction effects between Met source and SAA level for the carcass traits (Table 6). However, pigs which received diets supplemented with SAA at 120% presented better (*p* < 0.05) results of carcass weight (78.16 vs. 74.70 kg, *p* < 0.001) and of kg of lean meat (43.69 vs. 41.90, *p* < 0.001). Additionally, pigs fed diets supplemented with OH-Met presented better results for loin depth (58.37 vs. 55.21, *p* = 0.002).

**Table 5:** Individual liveweight of piglets fed with diets supplemented with DL-Methionine or OH-Methionine and sulfur amino acids at the recommended levels or above from 63 to 160 days old.

	SAA Level		Methionine Sources		SAA 100%		SAA 120%		SEM	*p*-Value		
	100%	120%	DL-Met	OH-Met	DL-Met	OH-Met	DL-Met	OH-Met		SAA Level	Met Source	Met Source × SAA Level
Days of age												
63	20.43	20.43	20.43	20.43	20.43	20.43	20.43	20.43	1.949	0.993	1.000	1.000
91	43.48	44.40	44.13	43.75	43.59	43.36	44.68	44.13	3.846	0.220	0.609	0.831
112	63.36 A	65.61 B	64.30	64.67	62.93	63.79	65.68	65.55	5.119	0.051	0.745	0.661
140	88.95 A	92.72 B	90.56	91.11	88.34	89.56	92.78	92.66	7.046	0.055	0.774	0.728
160	104.17 A	108.89 B	105.89	106.57	103.92	104.42	107.86	108.73	6.979	0.055	0.742	0.929

DL-Met: DL-methionine; OH-Met: OH-Methionine. Differences between means in the same column are indicated with A, B letters for *p* < 0.10.

## 4. Discussion

Overall, the performance results of the pigs, independent of the treatments applied, were below expectation. This behavior could be associated with the environmental temperature and the ingredients used in the experimental feeds. Pigs were under hot conditions and ingredients such as DDGS and wheat grain, which present higher levels of fiber [2] than a corn and soya meal-based diet, might have affected feed consumption and weight gain of the animals [16].

Performance data, carcass, and meat traits are often used to assess the bio-efficacy of amino acid supplements, especially in tropical conditions [17,18]. Throughout this study, the average temperatures were 27.12 ± 4.51 °C, well above the upper critical temperature for growing and finishing pigs (which is 23 °C). At these temperatures the pigs may present behavioral and physiological changes to maintain homeothermy. This has a metabolic cost [19] as it can change their patterns of nutrient utilization. On top of that, high temperatures can also alter the blood flow to the peripheral regions and can cause intestinal hypoxia, ATP depletion, oxidative stress, increased intestinal permeability, and apoptosis [20,21,22]. The increase in intestinal permeability can enable the absorption of endotoxins at the expense of the adequate use of nutrients [23], including methionine, thus impairing performance.

When the average sulphur amino acids intake (Table 3) and average feed intake (Table 4) obtained in this experiment were compared to those suggested by PIC genetic manual [24], results confirmed that not only in relative terms, but also in absolute terms (g/day), the sulphur amino acids intake (100 or 120% SAA) in each phase of the feeding program were above that of the genetic recommendation. The PIC daily feed intake (kg/day) and the absolute methionine and methionine + cystine consumption (g/day) for growing I, growing II, finishing I, and finishing II are, respectively, 1.360, 4.7, 9.4, 1.940, 5.8, 11.5, 2.540, 6.4, 12.9, 2.59, 59, and 11.9. This suggests that feed intake was not the main reason impairing weight gain of the animals; rather, heat stress played an important role in performance.

The performance results (Table 4) were not different for methionine sources. There are very few studies that assess the influence of the different methionine sources in growing and finishing pigs under heat stress. Broilers, however, have been studied extensively under heat stress and the results show that supplementation with methionine seems to reduce the negative effects of high temperatures, especially when using it in OH-Met form [15,25]. Willemsen et al. [15] reported that OH-Met supplementation resulted in higher growth in broilers under heat stress than DL-Met.

Increased supply of TSAA results in improved performance in other challenging conditions as well. In cases of LPS-induced stress, prolonged supplementation of methionine in post-weaning diet can reduce the adverse effects due to its anti-inflammatory and antioxidant properties [13]. This theory can explain the better performance results obtained with the highest levels of TSAA, considering thermal stress as a challenge present in this study.

In relation to greater TSAA supplementation, it is recognized that this procedure can affect the amino acid composition of tissue proteins, the glutathione-redox balance, and the muscle fiber type [8,13]. According to Conde Aguilera et al. [6], the reduction in SAA supply (by 36% of the amount recommended by the NRC 1998) decreases protein retention and daily weight gain and leads to an increase in lipid retention. These findings are similarly to Zhang et al. [26], who working with sows, demonstrated that, when supplying the females with methionine 25% over the NRC 2012 recommendation, the sows produced more and better-quality milk, leading to better performance of their litters. Other studies have shown the beneficial effects of methionine supplementation, especially in OH-Met form, on late gestating and lactating sows as well as on the performance of their litters [13,26].

Another hypothesis to explain the better weight gain of animals fed with higher amounts of TSAA would be the genetic potential of the animals used in our study, which might have presented greater muscle deposition and, consequently, higher amino acid requirements than the recommendation of the Brazilian Table. This hypothesis is supported by van Milgen and Dourmad [27], who find that amino acid requirements change as genotypes improve. On the other hand, the standard AA content in finishing diets increases the feed intake and results in an extra amount of energy eaten, and the pig might gain fat, with consequent changes of worse of carcass quality and market value [28,29].

It is interesting to note that the increase in weight gain of pigs was accompanied by an increase in feed consumption in the grower II and finisher I phases, which demonstrates that the animals that received diets with lower TSSA content (level 100%) consumed feed with a possible imbalance of amino acids, not meeting the concept of ideal protein, a fact that limits the ad libitum consumption of feed by animals [30,31]. In the grower phase I, it is possible that the animals still young (60 to 91 days) did not have the physiological capacity to increase their voluntary feed intake, even when consuming feeds with a higher content of amino acids (sulphur amino acids).

OH-Met determined a greater L. dorsi muscle depth compared DL-Met use (Table 6) for the carcass parameters, and the effects of using the higher dose of methionine supplementation were also significant (*p* < 0.05), with a positive impact on the weight and the amount of lean meat in the carcass.

OH-Met, because of its organic acid characteristics, is totally absorbed in pigs by the last section of the duodenum [12] and is not totally dependent on active modes of absorption, as DL-Met or L-Met are. Another possible enteric benefit of the use of OH-Met in pig diets, suggested by Apajalahti et al. [32], is that OH-Met, being an organic acid with a pKa value of 3.53, could theoretically modulate the gut microbiota and improve the nutrient absorption.

Our carcass results are also in line with the findings of Pillai et al. [33], who suggest that methionine supplementation has a more significant impact on the final weight and the carcass weight than on the carcass yield, and those found by Conde Aguilera et al. [7], whose results showed higher carcass weight for pigs that were fed higher doses of SAA. Their research, however, did not show any differences for carcass quality parameters.

These results are not similar to the findings of Yuan et al. [34], who observed that when higher doses of TSAA were supplemented (25% above the 2012 NRC recommendations) and different methionine sources were used, neither live weight nor carcass quality parameters were affected, although there was a modification in lipid metabolism when OH-Met was used.

When evaluating increasing doses of DL-Met, Santos et al. [35] did not find any differences in the carcass parameters of growing and finishing pigs. Similarly, Lebret et al. [36] registered no impact of high doses of methionine supplementation (OH-Met) on performance parameters and carcass quality of finishing pigs. However, the authors found improvements in meat quality, in particular, pH and color.

Studies indicate that dietary supplementation of methionine under OH-Met form may improve blood flow and the net absorption of portal amino acids, providing further positive nutritional effects on pig performance [10,13]. Additionally, according to Zhang et al. [26], OH-Met has certain antioxidant properties that are able to boost the animals’ immune system and alleviate heat stress. The higher concentration of circulating plasma taurine in pigs fed OH-Met compared to the DL-Met supplementation may [12] also bring insight to the better antioxidant properties of OH-Met. These mentioned qualities of OH-Met form, specially related to the net amino acids absorption [10,13], may support the better muscle depth results observed in our studies.

Heat stress is expected to cause an increased amount of fat at the expense of lean tissue in pork meat carcasses [37]. Thus, heat stress can alter the hierarchy of nutrient partitioning, regardless of the used nutritional plan [38]. However, the results observed in the present study suggest that higher doses of methionine might circumvent the detrimental effect of high temperatures in growing and finishing pigs, improving performance and carcass characteristics, and when using methionine in OH-Met form there is also an increase in loin depth.

## 5. Conclusions

In tropical climates an increased methionine supplementation of growing and finishing pigs provided better daily weight gain and final weight. In addition, high dosages of methionine provided heavier carcasses and more kg of lean meat. Animals supplemented with methionine in OH-methionine form presented better loin depth and similar performance and carcass traits compared with DL-methionine, showing OH-methionine potential as a source of this amino acid.

## Figures and Tables

**Table 2:** Analyzed dry matter, gross energy, and amino acids composition (as fed) of the experimental diets.

	Dry Matter, %	Gross Energy, kcal/kg	Total AA, %	Lys, %	Thr, %	Val, %	Met ^1^, %	Synthetic Added Met ^1^, %
Grower I (63–91 d old)								
100% SAA DL-Met	90.3	4600	16.6	0.97	0.80	0.86	0.44	0.07
100% SAA OH-Met	90.2	4610	16.8	1.01	0.75	0.74	0.47	0.08
120% SAA DL- Met	89.8	4610	16.9	0.95	0.70	0.74	0.54	0.13
120% SAA OH-Met	89.7	4590	15.6	0.99	0.69	0.68	0.52	0.14
Grower II (92–112 d old)								
100% SAA DL-Met	91.0	4630	16.3	0.99	0.69	0.73	0.41	0.07
100% SAA OH-Met	90.7	4620	14.8	0.88	0.74	0.66	0.39	0.06
120% SAA DL- Met	90.4	4620	16.3	0.91	0.78	0.82	0.48	0.11
120% SAA OH-Met	91.0	4560	15.1	0.92	0.76	0.68	0.45	0.11
Finisher I (113–140 d old)								
100% SAA DL-Met	90.0	4620	14.7	0.83	0.61	0.63	0.45	0.07
100% SAA OH-Met	90.4	4620	14.9	0.92	0.69	0.70	0.39	0.06
120% SAA DL- Met	92.7	4680	15.3	0.88	0.63	0.67	0.52	0.12
120% SAA OH-Met	91.1	4560	14.3	0.93	0.69	0.68	0.44	0.12
Finisher II (141–160 d old)								
100% SAA DL-Met	91.0	4590	13.9	0.83	0.62	0.62	0.37	0.05
100% SAA OH-Met	91.2	4590	14.5	0.83	0.64	0.65	0.38	0.04
120% SAA DL- Met	89.8	4570	14.0	0.84	0.63	0.62	0.45	0.10
120% SAA OH-Met	90.2	4550	14.5	0.89	0.64	0.66	0.42	0.09

^1^ Met equivalent. % of AA expressed from fresh matter.

**Table 3:** Calculated methionine and methionine consumption according to the SAA levels and phases.

		Consumption of Methionine (g/Pig/Day)					Consumption of Methionine + Cysteine (g/Pig/Day)				
		Grower I (63–91 d Old)	Grower II (92–112 d Old)	Finisher I (113–140 d Old)	Finisher II (141–160 d Old)	Total (63–160 d Old)	Grower I (63–91 d Old)	Grower II (92–112 d Old)	Finisher I (113–140 d Old)	Finisher II (141–160 d Old)	Total (63–160 d Old)
SAA level	100%	4.45 ^b^	6.29 ^b^	7.23 ^b^	6.13 ^b^	5.89 ^b^	8.76 ^b^	12,16 ^b^	13.96 ^b^	11.76 ^b^	11.42 ^b^
	120%	5.33 ^a^	8.11 ^a^	9.09 ^a^	7.55 ^a^	7.36 ^a^	9.26 ^a^	15.99 ^a^	17.66 ^a^	14.59 ^a^	14.44 ^a^
	*p*-value SAA level	0.000	0.000	0.000	0.000	0.000	0.018	0.000	0.000	0.000	0.000
	SEM	0.637	1.227	1.271	0.993	0.917	0.874	2.507	2.499	1.947	0.1848

Differences between means in the same column are indicated with superscript letters: a, b for *p* < 0.05;

**Table 4:** Performance of piglets fed with diets supplemented with DL-Methionine or OH-Methionine at the recommendations in sulfur amino acids or above from 63 to 160 days old.

	SAA Level		Methionine Sources		SAA 100%		SAA 120%		SEM	*p*-Value		
	100%	120%	DL-Met	OH-Met	DL-Met	OH-Met	DL-Met	OH-Met		SAA Level	Met Source	Met Source × SAA Level
Grower I (63–91 d old)												
Feed intake, kg/d	1.39	1.40	1.40	1.38	1.37	1.40	1.43	1.37	0.127	0.647	0.612	0.199
Weight gain, kg/d	0.77	0.80	0.79	0.77	0.77	0.76	0.80	0.79	0.078	0.074	0.53	0.795
FCR	1.81	1.76	1.78	1.79	1.78	1.83	1.77	1.74	0.094	0.127	0.76	0.227
Grower II (92–112 d old)												
Feed intake, kg/d	2.09 ^b^	2.25 ^a^	2.14	2.19	2.07	2.11	2.22	2.28	0.257	0.006	0.51	0.897
Weight gain, kg/d	0.95 ^a^	1.01 ^b^	0.96	0.99	0.92	0.97	1.00	1.01	0.104	0.026	0.26	0.617
FCR	2.21	2.23	2.24	2.20	2.25	2.17	2.22	2.24	0.14	0.664	0.46	0.241
Finisher I (113–140 d old)												
Feed intake, kg/d	2.49 ^B^	2.60 ^A^	2.51	2.58	2.48	2.51	2.55	2.64	0.275	0.087	0.485	0.766
Weight gain, kg/d	0.91 ^B^	0.97 ^A^	0.94	0.94	0.91	0.92	0.97	0.97	0.114	0.09	0.86	0.869
FCR	2.75	2.68	2.70	2.73	2.76	2.74	2.64	2.72	0.213	0.295	0.538	0.43
Finisher II (141–160 d old)												
Feed intake, kg/d	2.56	2.61	2.55	2.62	2.57	2.54	2.52	2.69	0.268	0.556	0.409	0.263
Weight gain, kg/d	0.85	0.86	0.85	0.86	0.87	0.83	0.84	0.89	0.116	0.581	0.833	0.2
FCR	3.04	3.03	3.01	3.07	2.98	3.10	3.03	3.04	0.231	0.88	0.381	0.489
Total (63–160 d old)												
Feed intake, kg/d	2.05	2.13	2.07	2.11	2.04	2.06	2.11	2.16	0.176	0.141	0.501	0.795
Weight gain, kg/d	0.86 ^b^	0.91 ^a^	0.88	0.89	0.86	0.87	0.90	0.91	0.064	0.03	0.633	0.95
FCR	2.37	2.34	2.35	2.36	2.36	2.37	2.33	2.36	0.072	0.363	0.554	0.646

DL-Met: DL-methionine; OH-Met: OH-Methionine. FCR: Feed Conversion Ratio. Differences between means in the same column are indicated with superscript letters: a, b for *p* < 0.05; A, B for *p* < 0.10.

**Table 6:** Averages of carcass weight (CW), backfat thickness (BT), loin depth (LD), % of lean meat (PLM), and kg of lean meat (KLM) according to treatments sources, doses, and animals’ sex.

	SAA Level		Methionine Sources		SAA 100%		SAA 120%		SEM	*p*-Value		
	100%	120%	DL-Met	OH-Met	DL-Met	OH-Met	DL-Met	OH-Met		SAA Level	Met Source	Met Source × SAA Level
CW (kg)	74.7 ^b^	78.16 ^a^	76.08	76.82	74.29	75.11	77.77	78.6	7.284	<0.001	0.434	0.636
BT (mm)	14.18	14.51	14.2	14.5	14.01	14.35	14.38	14.65	3.609	0.554	0.657	0.841
LD (mm)	56.46	57.04	55.21 ^b^	58.37 ^a^	54.56	58.35	55.82	58.4	7.303	0.582	0.002	0.49
PLM (%)	56.19	56	56.05	56.14	56.13	56.25	55.98	56.03	2.878	0.678	0.732	0.987
KLM (kg)	41.90 ^b^	43.69 ^a^	42.58	43.03	41.63	42.17	43.46	43.94	3.852	<0.001	0.361	0.676

DL-Met: DL-methionine; OH-Met: OH-Methionine. Differences between means in the same column are indicated with superscript letters: a, b for *p* < 0.05.

## Data Availability

The data presented in this study are available on request from the corresponding author.
