# Correction: da Silva et al. Increased Sulphur Amino Acids Consumption as OH-Methionine or DL-Methionine Improves Growth Performance and Carcass Traits of Growing-Finishing Pigs Fed under Hot Conditions. *Animals* 2022, *12*, 2159

**DOI:** 10.3390/ani13061099

**Published:** 2023-03-20

**Authors:** Caio Abércio da Silva, Cleandro Pazinato Dias, Marco Aurélio Callegari, Kelly Lais de Souza, José Henrique Barbi, Naiara Simarro Fagundes, Dolores I. Batonon-Alavo, Luciana Foppa

**Affiliations:** 1 Department of Animal Production, Center of Agrarian Science, State University of Londrina, Londrina 86057-970, PR, Brazil; 2 Akei Animal Research, Estrada Vicinal Fartura—Areias, Km 3|Três Saltos, Fartura 18870-000, SP, Brazil; 3 Adisseo France S.A.S., 10, Place du Général de Gaulle, 92160 Antony, France

## Error in Table

In the original publication [1], there was a mistake in column “SEM” of Table 5. The numbers originally presented were incorrect. They should have been presented with a decimal point (i.e., 1.949, 3.846, 5.119, 7.046, and 6.979). Below is shown the column with the correct values.
**SEM**1.9493.8465.1197.0466.979

Additionally, in the Table 5, the column “Methionine Sources”, row 4, shown below, had two incorrect numbers: 9.056 and 9.111.


**Methionine Sources**

**DL-Met**

**OH-Met**
20.4320.4344.1343.7564.3064.679.0569.111105.89106.57

The numbers should be 90.56 and 91.11.

Another mistake was presented in Table 6, where we changed the values of the parameters of the columns referring to SAA Level and Sources of Methionine by the values of the parameters of the columns referring to SAA 100% and SAA 120%.

Below are shown both the incorrect values and the corrected values, where the bold numbers are the correct values.
**SAA Level****Methionine Sources****SAA 100%****SAA 120%****100%****120%****DL-Met****OH-Met****DL-Met****OH-Met****DL-Met****OH-Met**74.2975.1177.7778.676.0876.8274.70 ^b^78.16 ^a^14.0114.3514.3814.6514.214.514.1814.5154.5658.3555.8258.455.21 ^b^58.37 ^a^56.4657.0456.1356.2555.9856.0356.0556.1456.195641.6342.1743.4643.9442.5843.0341.90 ^b^43.69 ^a^


**SAA Level**

**Methionine Sources**

**SAA 100%**

**SAA 120%**

**100%**

**120%**

**DL-Met**

**OH-Met**

**DL-Met**

**OH-Met**

**DL-Met**

**OH-Met**

**74.70 ^b^**

**78.16 ^a^**

**76.08**

**76.82**

**74.29**

**75.11**

**77.77**

**78.6**

**14.18**

**14.51**

**14.2**

**14.5**

**14.01**

**14.35**

**14.38**

**14.65**

**56.46**

**57.04**

**55.21 ^b^**

**58.37 ^a^**

**54.56**

**58.35**

**55.82**

**58.4**

**56.19**

**56**

**56.05**

**56.14**

**56.13**

**56.25**

**55.98**

**56.03**

**41.90 ^b^**

**43.69 ^a^**

**42.58**

**43.03**

**41.63**

**42.17**

**43.46**

**43.94**


It is important to note that the *p* values referred to in the article are the correct values. Therefore, there was no need to make any changes. Furthermore, the accompanying discussions provided in the text are also correct, so it is not necessary to change anything other than the values listed above.

The authors state that the scientific conclusions are unaffected. This correction was approved by the Academic Editor. The original publication has also been updated.
